# Allelic Diversity of the *Plasmodium falciparum* Erythrocyte Membrane Protein 1 Entails Variant-Specific Red Cell Surface Epitopes

**DOI:** 10.1371/journal.pone.0016544

**Published:** 2011-01-27

**Authors:** Inès Vigan-Womas, Micheline Guillotte, Alexandre Juillerat, Cindy Vallieres, Anita Lewit-Bentley, Adama Tall, Laurence Baril, Graham A. Bentley, Odile Mercereau-Puijalon

**Affiliations:** 1 Institut Pasteur, Unité d'Immunologie Moléculaire des Parasites, Paris, France; 2 CNRS URA 2581, Paris, France; 3 Institut Pasteur, Unité d'Immunologie Structurale, Paris, France; 4 CNRS URA 2185, Paris, France; 5 Unité d'Epidémiologie des Maladies Infectieuses, Institut Pasteur, Dakar, Sénégal; Agency for Science, Technology and Research - Singapore Immunology Network, Singapore

## Abstract

The clonally variant *Plasmodium falciparum* PfEMP1 adhesin is a virulence factor and a prime target of humoral immunity. It is encoded by a repertoire of functionally differentiated *var* genes, which display architectural diversity and allelic polymorphism. Their serological relationship is key to understanding the evolutionary constraints on this gene family and rational vaccine design. Here, we investigated the Palo Alto/VarO and IT4/R29 and 3D7/PF13_003 parasites lines. VarO and R29 form rosettes with uninfected erythrocytes, a phenotype associated with severe malaria. They express an allelic Cys2/group A NTS-DBL1α_1_ PfEMP1 domain implicated in rosetting, whose 3D7 ortholog is encoded by PF13_0003. Using these three recombinant NTS-DBL1α_1_ domains, we elicited antibodies in mice that were used to develop monovariant cultures by panning selection. The 3D7/PF13_0003 parasites formed rosettes, revealing a correlation between sequence identity and virulence phenotype. The antibodies cross-reacted with the allelic domains in ELISA but only minimally with the Cys4/group B/C PFL1955w NTS-DBL1α. By contrast, they were variant-specific in surface seroreactivity of the monovariant-infected red cells by FACS analysis and in rosette-disruption assays. Thus, while ELISA can differentiate serogroups, surface reactivity assays define the more restrictive serotypes. Irrespective of cumulated exposure to infection, antibodies acquired by humans living in a malaria-endemic area also displayed a variant-specific surface reactivity. Although seroprevalence exceeded 90% for each rosetting line, the kinetics of acquistion of surface-reactive antibodies differed in the younger age groups. These data indicate that humans acquire an antibody repertoire to non-overlapping serotypes within a serogroup, consistent with an antibody-driven diversification pressure at the population level. In addition, the data provide important information for vaccine design, as production of a vaccine targeting rosetting PfEMP1 adhesins will require engineering to induce variant-transcending responses or combining multiple serotypes to elicit a broad spectrum of immunity.

## Introduction

In sub-Saharan Africa, the main burden of *Plasmodium falciparum* malarial disease affects children and adolescents, while older subjects usually experience asymptomatic infections. This is thought to reflect the gradual acquisition of immunity by cumulated exposure to successive episodes of malaria caused by diverse parasite strains [1] and antigenic variants [2], [3]. A major contributor to parasite diversity is the *Plasmodium falciparum* erythrocyte membrane protein 1 (PfEMP1) variant adhesin that the parasite inserts into the membrane of the erythrocyte in which it develops. Surface expression of PfEMP1 bestows on the infected red blood cell (iRBC) the capacity to cytoadhere to host cells [4], a characteristic of this *Plasmodium* species and considered as an important contributor to *falciparum* pathology. PfEMP1 is encoded by a family of ∼60 *var* genes, each of which codes for a protein displaying specific binding and serologic characteristics. Successive expression of distinct *var* genes by clonal antigenic variation is a strategy used by the parasite to escape the host immune response and to establish a persistent infection, thereby optimising transmission.

PfEMP1 is a multi-modular adhesin, with an extracellular binding region consisting of a variable number of different (five types) Duffy-binding-like (DBL) and (three types) cysteine-rich interdomain region (CIDR) adhesion domains, and a more conserved cytoplasmic tail [5]. Diversity of PfEMP1 occurs both within and between genomes. Within each genome, *var* paralogs (with the exception of *var3*) differ in sequence, number of modules and flanking regulatory sequences, and are classified into groups A, B, and C, and B/A and B/C hybrid groups. Most *var* genes are located in the unstable sub-telomeric regions of the chromosomes and gene recombination during both meiosis and mitosis between these paralogs is an important mechanism of repertoire diversification [6], [7], even though recombination is constrained to occur within the same group [8], [9]. At the population level, the overall structuring into groups is conserved but *var* repertoires differ in their mosaic arrangement within individual modules, contributing to extensive sequence (allelic) diversity of the domain orthologs. Chimerism is particularly marked in *var* genes from group A (also called UpsA), whose expression tends to be associated with severe malaria and with infection in young children with a poorly developed antibody response to the erythrocyte surface antigens [10], [11], [12], [13]. Whole genome comparison has shown that domain arrangement and association differ in the various group A paralogs from different repertoires, although individual domains have allelic forms [14]. High sequence diversity has been observed in an analysis of UpsA-associated DBLα tags from a global collection of parasites [15].

Factors regulating variant antigen diversification remain unclear but likely include a trade-off between diversifying antibody-driven selection and purifying function-constrained selection. Longitudinal follow-up studies indicate that malaria episodes tend to be caused by parasites expressing iRBC surface variants corresponding to gaps in the acquired antibody repertoire, suggesting negative selection by pre-existing immune responses [3], [16].

To gain insight into the immunological consequences of chimerism and allelic polymorphism, we compared the antibody response to a set of monovariant parasite lines that display an allelic form of a PfEMP1 adhesion domain. We chose to study the Palo Alto 89F5/VarO [17] and the IT4/R29 variant [18], which both express orthologous NTS-DBL1α_1_ adhesion domains mediating rosetting (i.e., binding uninfected red cells), a cytoadherence phenotype associated with severe malaria in African children [19], [20], [21], [22]. In addition, we identified PF13_0003 as carrying an orthologous adhesion domain in the 3D7 genome [23] and developed a monovariant 3D7/PF13 line that also formed rosettes. Apart from the NTS-DBL1α_1_ domain, homology between the downstream domains of the three PfEMP1 variants is modest, allowing to explore the consequence of allelic polymorphism of the adhesion domain associated with non-orthologous domains.

We set out to analyse the serological relationships between the three NTS-DBL1α_1_ adhesion domains, on one hand, and between the iRBC surfaces of the three lines, on the other, to study how allelic diversity impacts on antigenicity. We produced a soluble recombinant NTS-DBL1α_1_ adhesion domain from each variant. A prerequisite for the analysis of the iRBC surface serotype is the use of monovariant cultures in which all parasites express the same PfEMP1 variant. This had been achieved for VarO by positive selection using variant-specific antibodies raised to the recombinant NTS-DBL1α_1_ [17]. Using the same strategy, we have now developed monovariant IT4/R29 and 3D7/PF13 parasite lines, which both formed rosettes. Mouse antibodies elicited by each recombinant NTS-DBL1α_1_ domain cross-reacted with their allelic forms by ELISA but displayed strictly variant-specific iRBC surface reactivity and variant-specific rosette disruption. Analysis of naturally acquired antibodies from humans exposed to *P. falciparum* living in Dielmo, Senegal, showed positively correlated ELISA reactivities to the recombinant NTS-DBL1α_1_ domains. Here also, however, reactivity with the iRBC surface was variant-specific and the age-acquisition pattern differed for the three variants. Absorption of sera with the recombinant domain showed that the anti-NTS-DBL1α_1_ antibodies represented a large fraction of the iRBC surface reactivity. Taken together, these data demonstrate that the related rosette-forming parasites display variant-specific iRBC-surface epitopes. Thus, PfEMP1 allelic variation causes antigen diversification and participates in immune evasion at the population level. In addition, these data provide important information for vaccine development since a strategy targeting rosetting will need either to combine multiple serotypes or to engineer the antigen so as to broaden specificity.

## Results

### NTS-DBL1α_1_-VarO orthologs in the IT4 and 3D7 genome

**Figure 1:**
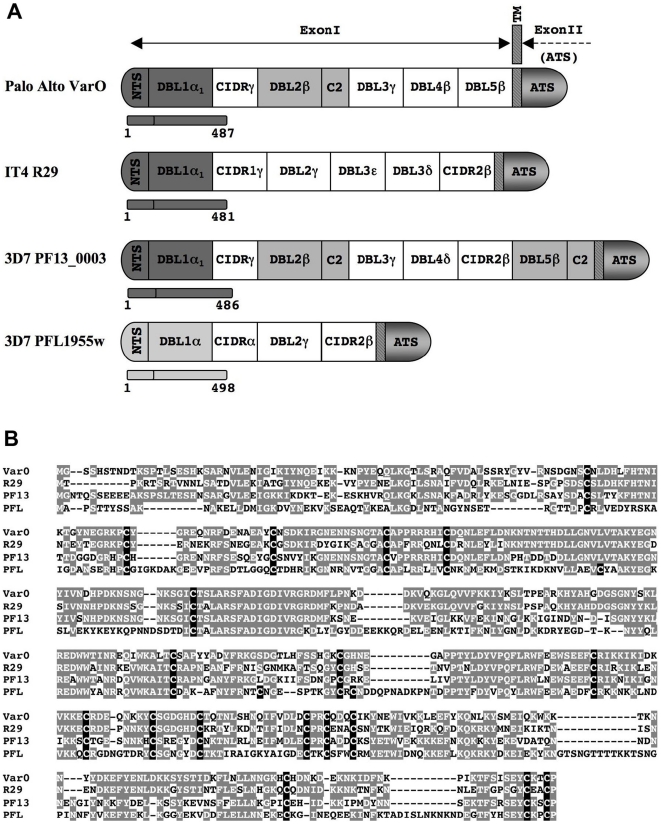
Domain organisation of the PfEMP1 proteins and sequence comparison of the NTS-DBL1α domains. (A) Schematic domain organisation of the Palo Alto/varO, IT4/R29, 3D7/PF13_0003 and 3D7/PFL1955w PfEMP1 proteins. Regions corresponding to the recombinant NTS-DBL1α domains are indicated by bars, with the N- and C-terminal residue numbers giving sequence limits. Domains in dark grey have >50% sequence identity, shaded DBLβ domains have ∼38% identity; DBL1α-PFL1955w is shown in light grey (29–33% identity). (B) The NTS-DBL1α sequences aligned as described in the Materials and Methods. Cysteine residues are shown in white on a black background and residues with 50% or more sequence identity are shown in white on a grey background.

In the 3D7 [23] and IT4 genomes [14] there are no *var* genes with a similar gene architecture and a high sequence identity score across the entire gene length, although the best identity scores for individual domains were found in the subset of group A genes [17]. Since the DBL1α_1_ domain mediates rosetting, we focussed further work on genes harbouring the orthologous domain, namely PF13_0003 (hereafter called PF13) and R29 (*IT4var9*) (hereafter called R29) from in the 3D7 and IT4 genomes, respectively. Both R29 and PF13 belong to the group A/UpsA *var* genes with a typical PoLV Group 1 (2 cysteines/MFK*) sequence signature [13], [24]. Pairwise identity between the domains is close to 60% across the NTS-DBL1α_1_ protein sequence. The downstream protein architecture of VarO, R29 and PF13 differs in the number and type of DBL domains, and the presence of a second CIDR domain in R29 and PF13 that is absent in VarO (Fig. 1). The VarO domains downstream from the NTS-DBL1α_1_ have limited homology with the downstream domains of R29 and PF13 (<30%), apart from the DBL2β and DBL5β domains of PF13 (38% identity) (Figure S1). Thus, VarO, R29 and PF13 display an allelic form of the NTS-DBL1α_1_ domain combined with non-allelic forms of partner domains.

### Production of R29 and PF13 NTS-DBL1α_1_ recombinant domains and mouse antisera

A recombinant, soluble NTS-DBL1α_1_ domain was produced in *E.coli* for R29 and PF13 with a C-terminal His-tag. Protein purity was checked by SDS-PAGE analysis under reducing and non-reducing conditions and immunoblotting. The proteins migrated at the expected molecular mass (Figure S2). N-terminal sequencing identified a single sequence (data not shown), confirming protein purity. Circular dichroism spectra in the near and far UV regions indicated that the proteins were properly folded and N-terminal sequencing of trypsinised fragments was consistent with the protein being produced with the native conformation (data not shown), as shown for the VarO domain [25].

**Figure 2:**
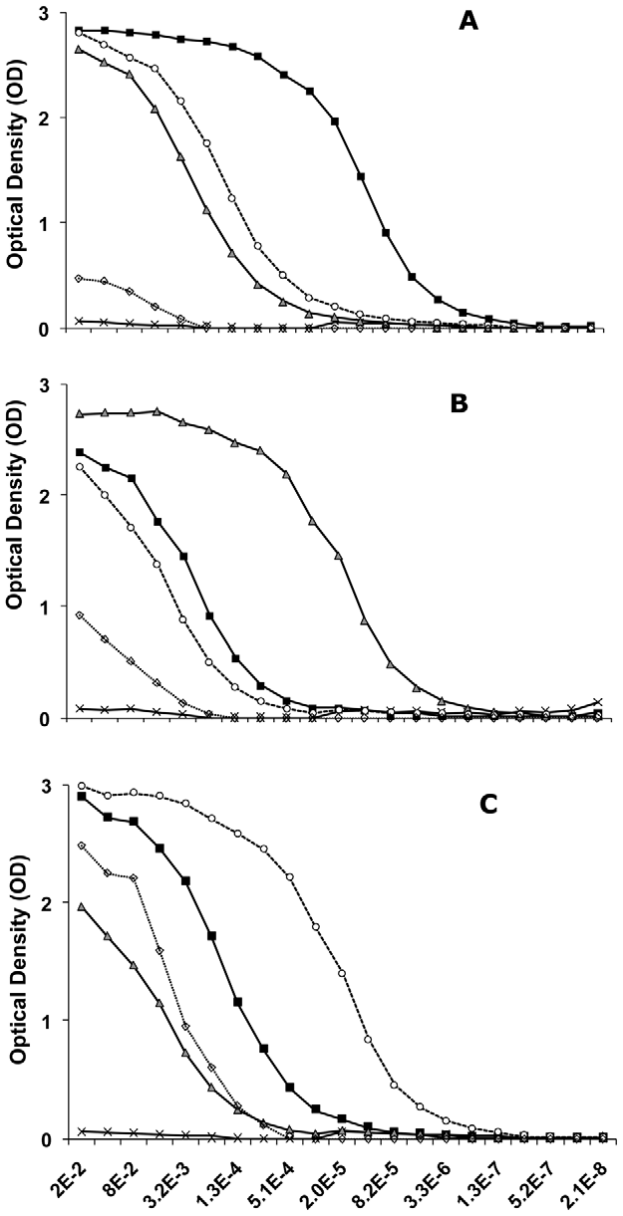
ELISA titration curves of mouse sera against the NTS-DBL1α recombinant domains. Serial two-fold dilutions of mouse anti-VarO (A), anti-R29 (B), anti-PF13 (C) polyclonal sera (initial dilution 1/200) tested by ELISA against the recombinant domains. The plates were coated with the VarO (squares), R29 (triangles), PF13 (open circles) and PFL1955w (diamonds) domains; (x) indicates pre-immune sera.

The R29 and PF13 proteins elicited a consistent, homogeneous and strong response, with high ELISA titres in outbred mice (N = 6 per antigen). The endpoint ELISA titre to the immunogen was >6×10^−7^ (Fig. 2), similar to the titre elicited by the VarO recombinant domain [25].

### Mouse anti-NTS-DBL1α_1_ antibodies cross-react in ELISA with the allelic recombinant domains

Cross-reactivity of the various mouse sera was analysed on the recombinant VarO, R29 and PF13 domains by ELISA and compared to the reactivity with a domain from a paralog from a distinct PoLV of DBL1α. We chose NTS-DBL1α PFL1955w, encoded by an UpsB/C type paralog, with a Cys4, group 4 DBL1α domain and displaying 32–33% identity with the VarO, R29 and PF13 domains (Figure S1). The recombinant PFL1955w domain was produced using a similar strategy and its recognition by the various antisera was studied in parallel with the other proteins.

The anti-NTS-DBL1α_1_-VarO sera cross-reacted with the R29 and PF13 alleles, with 30- and 16-fold reduction of the 95% titre, respectively; reaction with PFL1955w was quite modest as both signal and titre were low (Figures 2A and S3). Importantly, this was observed with sera raised to both the baculovirus and the *E. coli* NTS-DBL1α_1_-VarO proteins (data not shown). Cross-reactivity was not due to reaction with the His-tag present in the VarO, R29 and PF13 recombinant proteins, as the sera failed to react with e.g. the His-tagged CIDR-VarO construct (and *vice versa*, data not shown). The anti-R29 sera cross-reacted with the VarO and PF13 alleles with a 30- and 80-fold reduced 95% titre, respectively, and reacted poorly with PFL1955w (Figure 2B). The anti-PF13 sera cross-reacted more efficiently with VarO than with R29 (16- and 45-fold reduction of the 95% titre, respectively) (Figure 2C). They reacted with PFL1955w with a higher maximum value than the antisera raised to VarO or R29, although with low 50% and 95% titres.

### Establishment of monovariant cultures of R29 and PF13

**Figure 3:**
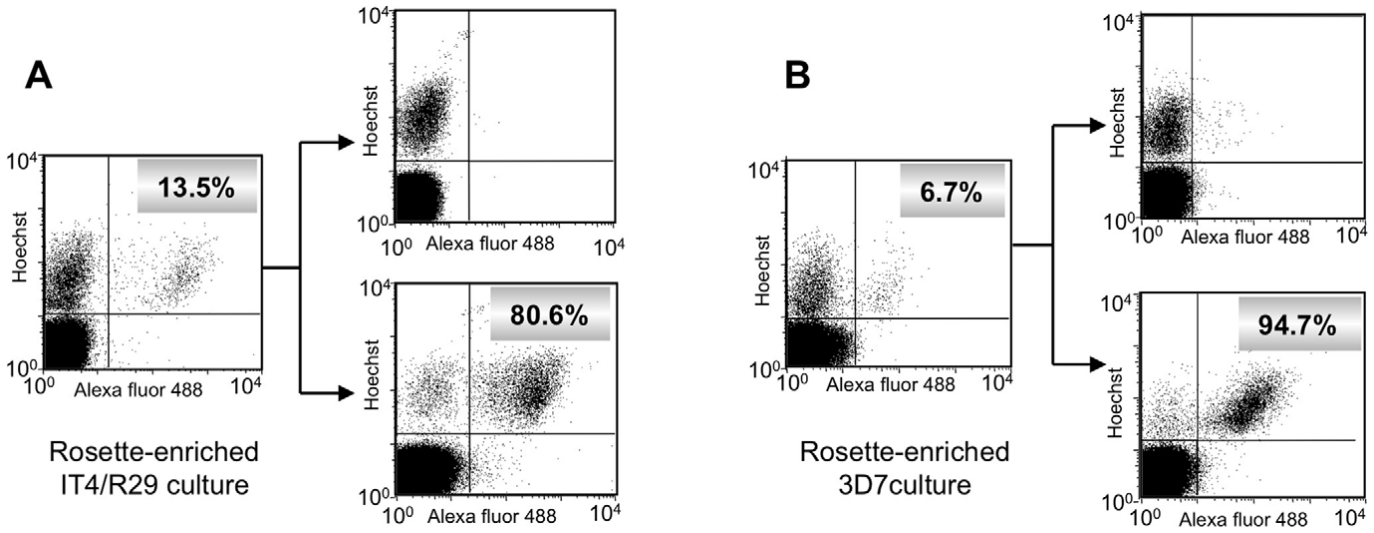
Isolation of monovariant cultures of IT4/R29 and 3D7/PF13_0003. Rosette-enriched IT4/R29 (A) and 3D7/PF13 (B) cultures were incubated with polyclonal sera raised against recombinant domains and were isolated by cell sorting (see Materials and Methods). The left-hand panels show surface positivity rates of the initial rosette-enriched cultures and the right-hand panels show the surface reactivity of the negative (upper) and positive (lower) sorted populations. The x and y axes show Log10 fluorescence.

To investigate iRBC surface reactivity, we developed monovariant cultures of R29 and PF13, with >95% iRBC displaying the PfEMP1 in question. Initially, the anti-NTS-DBL1α_1_-R29 polyclonal mouse antibodies reacted with less than 15% of the trophozoite/schizont stages from a rosette-enriched IT4/R29 culture in which the rosetting phenotype had been maintained by weekly enrichment (Figure 3A). This heterogeneity reflected antigenic switching to other rosette-forming parasites that no longer expressed R29. To enrich with parasites expressing R29, we isolated the sub-population of anti-R29-positive iRBCs from the rosette-enriched culture by cell sorting. This allowed to establish an *in vitro* single variant culture of R29 in which a vast majority of iRBC expressed the R29 protein.

To develop a single variant PF13 model, we hypothesised that the PF13_0003 gene encoded a PfEMP1 mediating the formation of rosettes, like its orthologs in the IT4 and Palo Alto lines. We performed a weekly enrichment of rosette-forming parasites, starting from a standard long-term 3D7 culture. After 10 weeks of rosette-enrichment, a rosetting 3D7 culture was established. As hypothesised, a fraction of these rosette-forming parasites tested positive by flow cytometry for surface expression using the polyclonal mouse anti-NTS-DBL1α_1_-PF13antibodies (Figure 3B). Cell sorting from the multi-variant rosette preparation using the polyclonal mouse sera selected the sub-population recognised by the anti-NTS-DBL1α_1_-PF13 antibodies, allowing to establish a monovariant PF13 culture, with 95% or more of the trophozoites/schizonts staining positive and forming rosettes (Figure S4).

Further characterisation of the R29 and PF13 monovariant cultures was carried out by RT-PCR, with mRNA extracted from mature trophozoites. Analysis of the cDNA sequences obtained for each culture perfectly matched the published sequence of R29 and PF13, respectively, indicating that indeed, the R29- and PF13-selected parasites expressed the corresponding PfEMP1 adhesin.

Subsequently, monoclonal antibodies (mAbs) were isolated from animals immunised with the R29 or PF13 recombinant domain and used bi-monthly for routine enrichment of R29-expressing IT4 cultures or PF13-expressing 3D7 parasites following the procedure described for VarO [17].

### Absence of surface cross-reactivity of mouse sera raised to the recombinant domains

**Figure 4:**
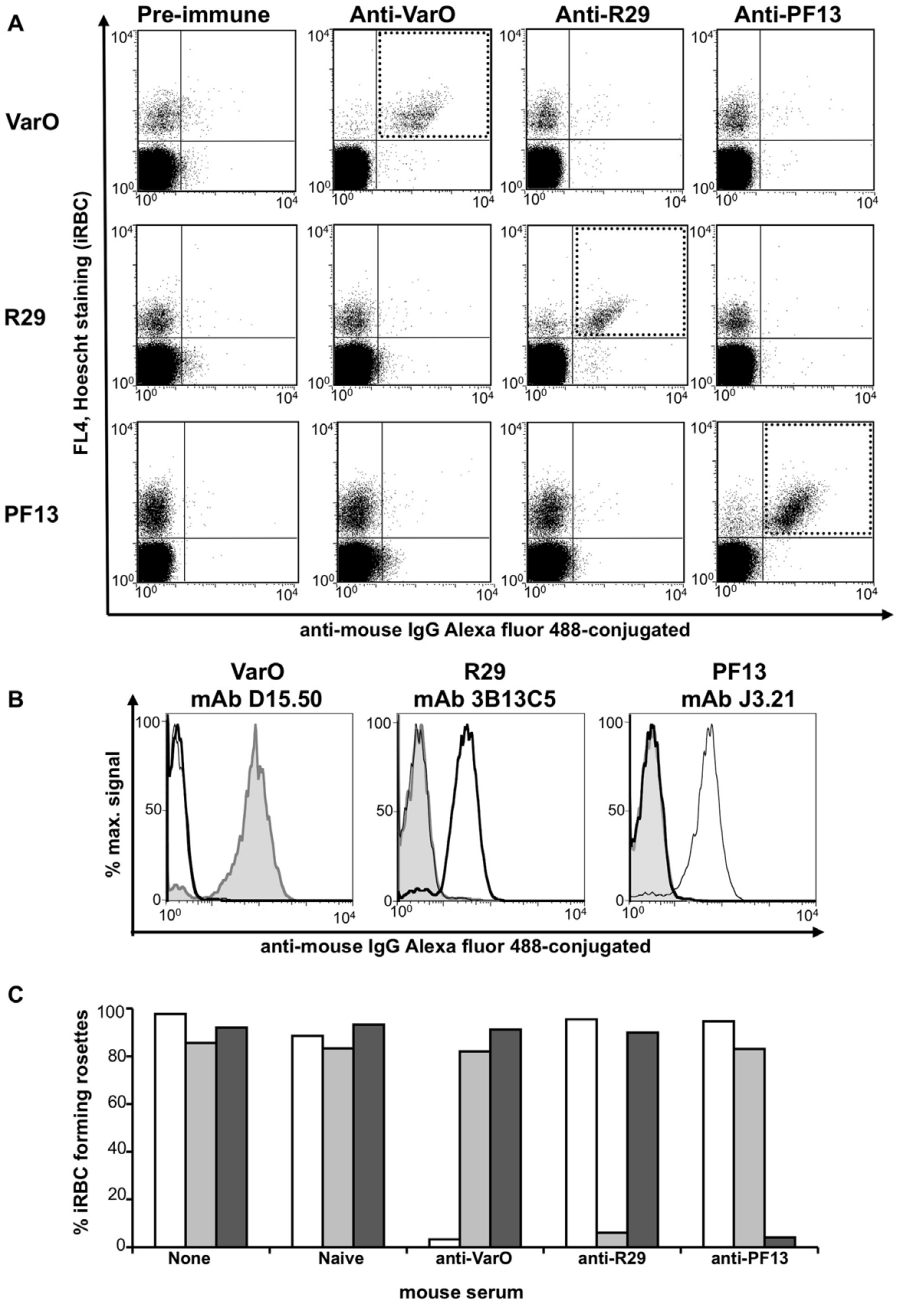
Variant-specific iRBC surface serotypes of Palo Alto/VarO, IT4/R29 and 3D7/PF13. (A) Surface immunofluorescence: monovariant lines were incubated with mouse sera raised to the recombinant domain and analysed by FACS. Rows and columns show parasite lines and sera, respectively, as indicated. The x and y axes show Log10 fluorescence. (B) Variant-specific surface reactivity of the mAbs raised to the recombinant domains with the monovariant lines analysed by FACS. In each panel, the shaded area, the thick line and the thin line indicate reactivity with Palo Alto/VarO, IT4/R29 and 3D7/PF13, respectively. (C) Variant-specific rosette disruption. Monovariant cultures were incubated with mouse serum as indicated and the fraction of mature stages forming rosettes was monitored. White, light grey and dark grey bars indicate Palo Alto/VarO, IT4/R29 and 3D7/PF13, respectively.

The iRBC-surface reactivity of the various mouse anti-NTS-DBL1α_1_ antibodies was analysed using the VarO, R29 and PF13 monovariant cultures. Although each antiserum reacted with its cognate iRBC-surface, no cross-reactivity was observed with the heterologous cultures expressing an allelic form of the NTS-DBL1α_1_ domain (Figure 4A). This also held true when surface reactivity of mAbs raised to the individual recombinant domain was studied, as each reacted solely with the iRBC expressing the cognate PfEMP1 from which the immunising NTS-DBL1α_1_ was derived (Figure 4B). This allele-specific surface reactivity was further emphasised when rosette disruption was studied (Figure 4C). Each serum readily disrupted rosettes expressing the homologous antigen, but failed to disrupt the rosettes displaying an allelic form. These results indicate that the various NTS-DBL1α_1_ domains elicited allele-specific surface-reacting antibodies in the mouse.

### Elevated seroprevalence of domain-reactive and iRBC surface-reactive antibodies

**Figure 5:**
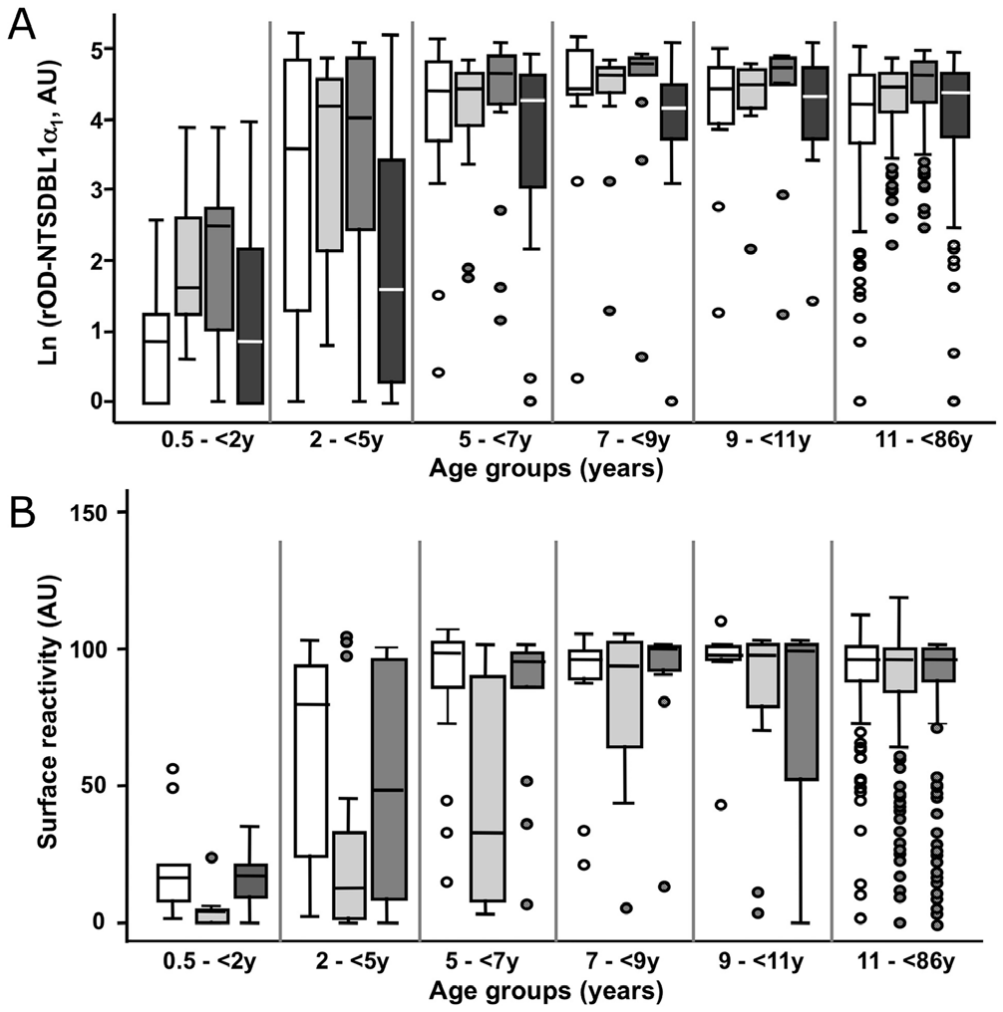
Seroprevalence for NTS-DBL1α domains and Palo Alto/VarO, IT4/R29 and 3D7/PF13 iRBC in Dielmo (Senegal). Seroprevalence to recombinant domains (A) and the iRBC surface (B) in Dielmo, Senegal, measured by ELISA and FACS, respectively. The boundaries of the boxes indicate the 25th and 75th percentiles, and the line in each box indicates the median. The whiskers indicate the 10th and 90th percentiles. The outlying dots show values exceeding the 10th and 90th percentiles. The number of persons by age group was 9, 23, 19, 14, 12 and 158 in the 0.5->2, 2-<5, 5-<7, 7-<9, 9-<11 and >11 years, respectively. Symbols used: open, VarO; light grey, R29; medium grey, PF13; dark grey, PFL1955w (in A only).

The prevalence of antibodies to the various NTS-DBL1α_1_ recombinant domains was studied in a community living in a malaria holoendemic area (Dielmo, Senegal). Serum samples of all individuals (0.5 to 85 year old) were analysed. The threshold for positive recognition was set from reactivity of non-immune French adults. Seroprevalence to the R29 and PF13 recombinant domains was very high, with 96.5% [95% confidence interval (CI) 94.07–98.9] and 99.1% [97.9–100] positive sera, respectively, compared to 87.7% [82.8–91.6] positive sera to VarO. As for VarO [17], R29- and PF13-NTS-DBL1α_1_ seroconversion was observed in the 2–5 y age group (Figure 5A). Seroprevalence to the group B/C PFL1955w domain was also quite high – at 95% [92.9–97.6] - although the age distribution differed and was shifted towards older children compared to the group A domains. The reactivities to the three group A domains were highly correlated with each other and moderately correlated to reactivity with PFL1955w (Table 1).

**Table 1:** Pairwise correlation of antibody reactivity to individual recombinant domains with reactivity to iRBC surface.

	recombinant NTS-DBL1α				iRBC surface		
	VarO	R29	PF13	PFL1955	VarO	R29	PF13
recombinant NTS-DBL1α							
VarO	1						
R29	.818#	1					
PF13	.784#	.815#	1				
PFL1955	.462#	.576#	.585#	1			
surface IFA							
VarO	.476#	.574#	612#	.462#	1		
R29	.520#	.611#	.663#	.512#	.632#	1	
PF13	.515#	.506#	.642#	.418#	.651#	.627#	1

# *P*<0.00001.

Pairwise comparisons are based on individual OD values determined by ELISA and mean fluorescence intensity determined by FACS (all age groups). Spearman correlation tests using STATA 9.2.

Prevalence of surface-reactive IgG was also high, with 81.7% [CI 76.4–86.9] and 87.1% [82.7–91.4] to the R29- and PF13-iRBC surface, respectively, but slightly lower than the 93.9% [89.7–96.4] previously observed for VarO with the same sera [17], shown here for comparison (Figure 5B). The prevalence of VarO- or PF13-surface-reactive antibodies rapidly increased with age, with more than 80% of children having acquired surface-reactive antibodies by the age of 5 y. The kinetics of acquisition for R29 was delayed by a few years, reaching its maximal value by the age of 9–11 y. Surface reactivity to the three variants was positively correlated. The correlation of iRBC surface reactivity with ELISA reactivity to the cognate recombinant domain was modest and did not differ from the correlation of reactivity with the allelic or outsider domain (Table 1).

**Figure 6:**
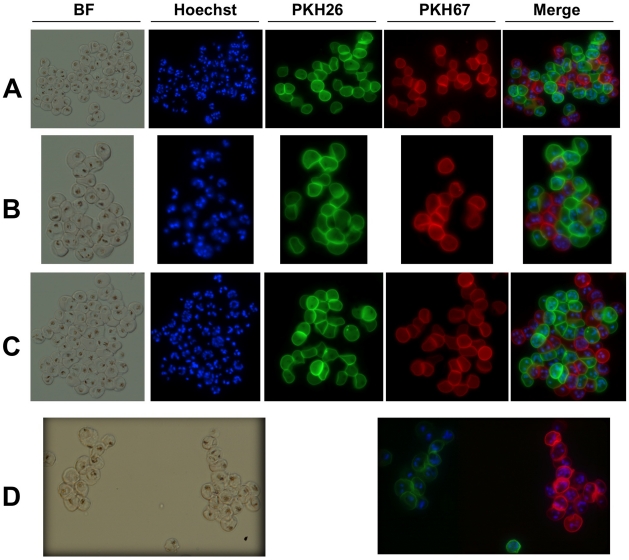
Mixed agglutination assay with Palo Alto/VarO, IT4/R29 and 3D7/PF13 monovariant lines. After rosette disruption with dextran sulphate, the RBC membrane was labelled with either PKH26 or PKH67, mixed in homologous or heterologous pairwise association, and incubated with human serum in the presence of dextran sulphate. Agglutination was examined using a fluorescence microscope. A representative example with an individual serum is shown. (A), (B) and (C) show mixed agglutinates of homologous PKH26- and PKH67-labeled Palo Alto/VarO, IT4/R29 and 3D7/PF13 associations, respectively, while (D) shows a typical absence of mixed agglutinates using heterologous association (shown is a PKH26-labeled Palo Alto/VarO and PKH67-labeled IT4/R29 association).

We next investigated whether naturally acquired antibodies to VarO, R29, and PF13 iRBC cross-reacted with the iRBC surface using the mixed agglutination assay [26]. For each rosetting variant, iRBC were magnetically selected to obtain >95% iRBC at mature stages and stained green or red with PKH57 or PKH26, respectively, before being incubated with human sera. To validate the assay, iRBCs of the same culture were differentially labelled with the two dyes, mixed and incubated in the presence of sera from non-immune individuals or from Dielmo villagers. No agglutinates were detected with non-immune sera (data not shown). VarO-, R29- or PF13-iRBC formed large mixed agglutinates when incubated with malaria hyper-immune sera (Figure 6). However, single-colour, variant-specific agglutinates were formed when using mixtures of differentially stained VarO and R29, VarO and PF13 or R29 and PF13 iRBC. Comparable results were obtained with pooled sera and individual sera (data not shown). Age of the serum donor did not influence serotype specificity, as single colour agglutinates were observed with sera from children as well as from adults.

**Figure 7:**
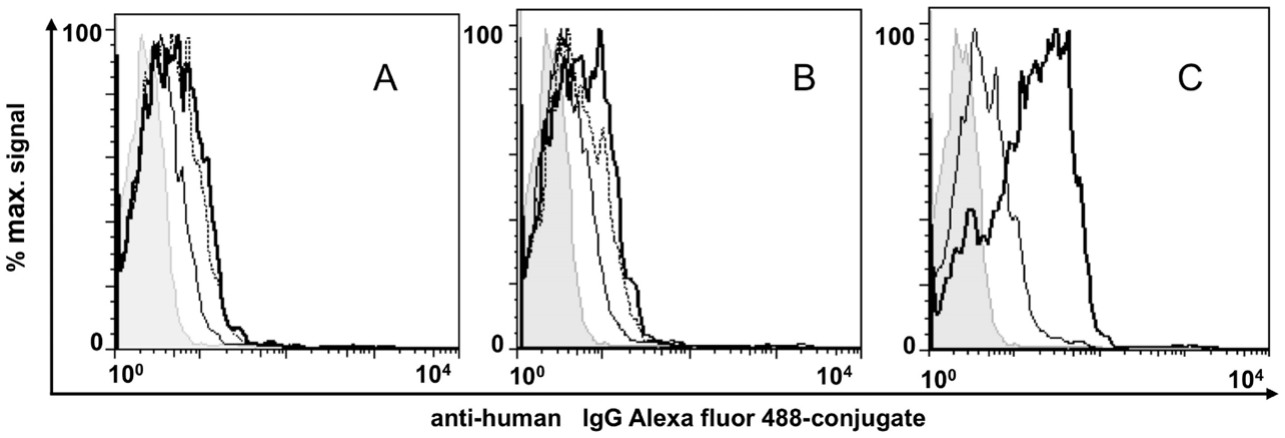
Residual VarO-iRBC surface reactivity after serum depletion of human sera using the recombinant VarO domain. FACS analysis of serum from adults (A and C) and from a 6 y old child (B) living in Dielmo, Senegal before and after antibody depletion. The grey area indicates fluorescence intensity distribution of non-immune French blood donors. The thick, dotted and thin lines indicate IFA distribution of non-depleted, control TALON-absorbed, recombinant VarO-depleted serum, respectively. The x axis shows Log10 fluorescence.

This indicates that naturally acquired antibodies to the *P. falciparum* variant surface are variant-specific. Reactivity with the iRBC surface is the sum of binding to multiple variable epitopes displayed by the various PfEMP1 domains and possibly by other variant surface antigens. To evaluate the contribution of anti-NTS-DBL1α_1_ antibodies to the overall iRBC surface-reactivity, sera were depleted of NTS-DBL1α_1_-reactive antibodies by immunoabsorption onto the recombinant antigen and tested for iRBC surface reactivity, mixed agglutination and rosette disruption. The mean fluorescence intensity was markedly reduced after depletion by NTS-DBL1α_1_ but was essentially unaffected by absorption with an irrelevant recombinant protein (Figure 7). This shows that anti-NTS-DBL1α_1_ antibodies constitute a large fraction of surface-reactive antibodies.

## Discussion

Allelic sequence diversity, chimerism and architectural diversity of the *var* repertoire, together, pose formidable obstacles to the study of immune responses acquired in endemic areas and to the rational design of vaccines aimed at preventing cytoadherence. Whether protective immunity is brought about by a broad repertoire of antibodies against polymorphic PfEMP1 epitopes that block reinfection, or by the slow acquisition of antibodies against conserved PfEMP1 epitopes, is a critical issue for understanding the evolutionary constraints on the *var* gene family and for designing interventions targeting this family of adhesins. The present work has aimed at obtaining insight into the immune responses mounted to PfEMP1 adhesins displaying an allelic form of a rosette-mediating adhesion domain, a cytoadherence phenotype commonly observed in severe malaria. We were particularly interested to determine whether naturally acquired antibodies to the surface-exposed PfEMP1 molecule in the three parasite lines cross-reacted and whether cross-reacting antibodies could be induced by immunising mice with the shared adhesion domain.

Monovariant clonal lines expressing a single iRBC surface serotype showed no evidence of antibody cross-reactivity of the surface-exposed PfEMP1 epitopes even though ELISA cross-reactivity to the natively folded recombinant domains was observed. Human sera formed single colour agglutinates, irrespective of the donor age. These non-overlapping surface serotypes indicate that allelic diversity and chimerism participate in strain and serotype structuring. Although surface serotypes differed, clear evidence of cross-reactivity with the recombinant NTS-DBL1α_1_ domains was observed in ELISA, suggesting that they belong to the same serogroup. The data further show that immunisation with the recombinant adhesion domain elicited variant-specific surface-reacting antibodies, implying that vaccine development will require either engineering an immunogen that induces variant-transcending responses or combining multiple serotypes in order to elicit a broad spectrum of antibodies to rosette-forming variants.

The three *var* genes studied possess the structural signatures of the small subgroup of group A/Cys2 type genes that has been more particularly associated with severe disease [11], [24] and/or with disease in poorly immune, young children [13]. The large allelic diversity of group A/Cys2 type *var* genes in the field [13], [15], [24] supports the view that they are under immune selection. The data presented here are consistent with this. Antibodies naturally acquired by the Senegalese villagers failed to cross-react with the iRBC surface of the heterologous rosette-forming variants irrespective of the combination used. A lack of surface serotype overlap was observed in all age groups, indicating that cumulative exposure results in accumulating distinct antibody specificities rather than developing broadly cross-reacting antibodies. The age-association of antibody distribution in the community and importantly, the non-overlapping surface serotypes, support the conclusion that allelic diversity is an immune escape and that antibodies participate in antigen diversification by selecting variant sequences and by structuring strains into separate serotypes. Nevertheless, we propose that serotypes include highly related sequences, as it is unlikely that parasites displaying the exact VarO, R29 or PF13_0003 sequences circulate in the village. In the absence of information on the local *var* repertoire, which we suppose is very large in view of the global diversity in Dielmo [27], we suggest that the observed antibody responses were elicited by parasites expressing genes presenting higher sequence identity than the three allelic domains studied here (i.e., >60% identity). The degree of homology needed to generate cross-reactive surface-reacting antibodies is unknown. One study in India reported the frequent occurrence of mixed-colour agglutinates with *P. falciparum* isolate pairs tested with convalescent-phase sera [28]. It is unclear why this was not observed in our study and in many studies with parasites and sera from other endemic areas [29], [30]. It is possible that mixed agglutination assays lack sufficient sensitivity to detect cross-reactive antibodies if these represent only a small fraction of the surface-reactive antibodies. However, even in the case of *var2csa*, which displays a limited population polymorphism [15], [31], surface serotypes do not fully overlap, as many human immune sera form single-colour, isolate-specific agglutinates with CSA-binding lines, with mixed agglutinates being infrequent and pan-agglutination quite rare [29], [31], [32]. Similarly, human mAbs reacting with PfEMP1-*var2csa* expressed by CSA-binding parasites seem to target determinants not present in all lines [33]. However, some antibodies elicited by *var2csa* or *var1csa* recombinant domains cross-reacted with the surface of heterologous lines [34], [35], [36], [37], [38], [39]. By contrast, neither the mouse antisera nor the mouse mAbs raised to the three recombinant rosetting domains cross-reacted with the heterologous iRBC surface and none were able to cross-disrupt rosettes. As the NTS-DBL1α_1_ is the most conserved domain between the three variants, the variant-specific surface reactivity could potentially be targeting the more distant PfEMP1 domains downstream or other variant surface antigens such as rifins or stevors. We can reasonably exclude this possibility, however, since the marked reduction of surface reactivity following absorption by the recombinant domain showed that antibodies to NTS-DBL1α_1_ accounted for a large fraction of surface seroreactivity.

**Figure 8:**
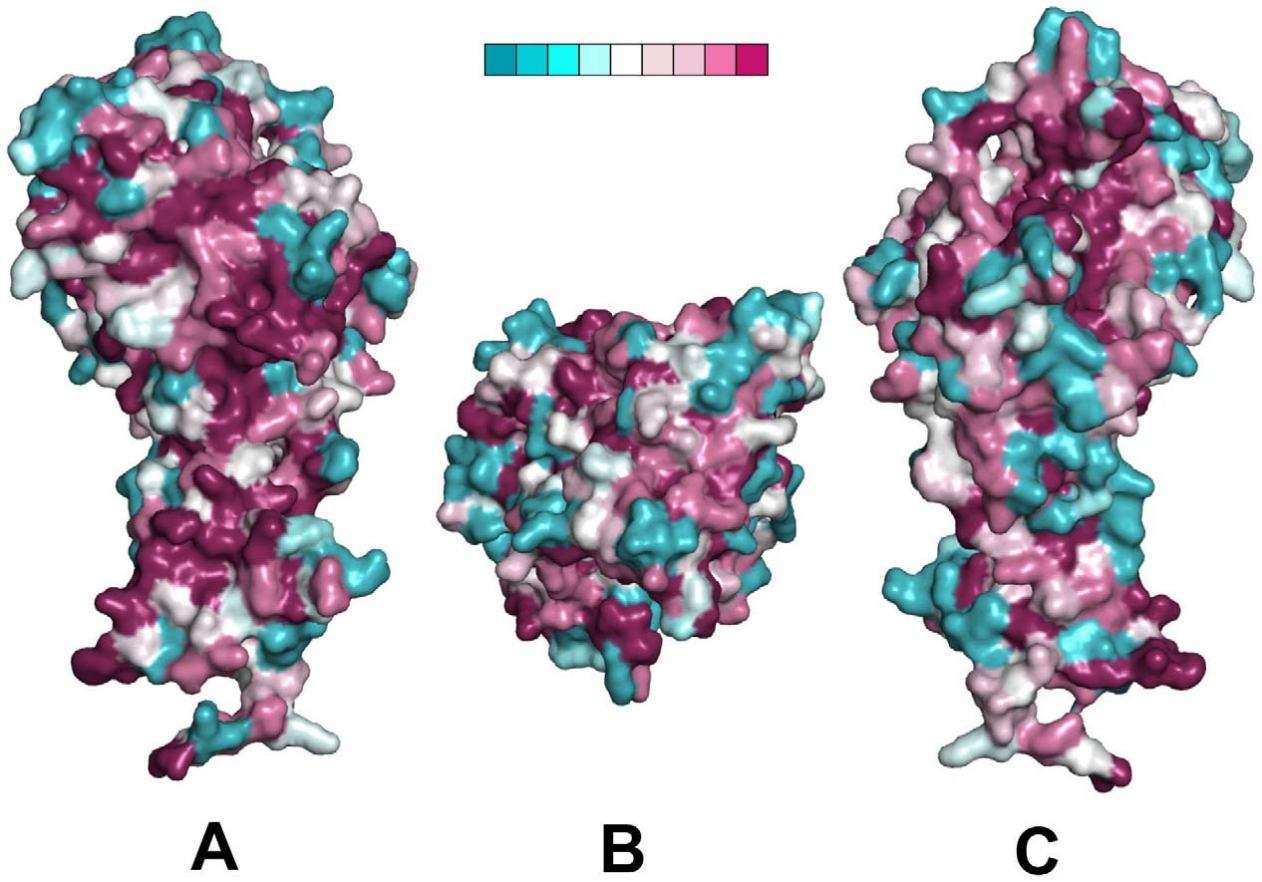
Sequence polymorphism of the three alleles mapped upon the NTS-DBL1α_1_ varO crystal structure. Three mutually orthogonal views of the surface of the NTS-DBL1α_1_-varO crystal structure with sequence polymorphism between the three alleles, VarO, R29 and PF13, colour-coded from blue (variable) to red (conserved - see the nine-point colour scale, insert). (A) and (C) are viewed with the C-terminus of the domain at the bottom and are rotated with respect to each other by 180° about a vertical axis. (B) is a perpendicular view, seen from above (A) and (C).

Mapping the polymorphic residues onto the recently determined crystal structure of NTS-DBL1α_1_-VarO (Juillerat et al, submitted) (Figure 8) revealed numerous surface-exposed polymorphic residues. These represent potential allelic variants of surface-displayed epitopes and most likely account for much of the specific surface seroreactivity of the human antibodies as well as of antibodies induced by the recombinant domain (including mAbs). Interestingly, however, the protein surface displays relatively conserved areas as well, which may be candidates for binding sites to heparin, which disrupts rosettes for all three variants [17], [40] (results not shown for PF13).

Another important lesson of the present work is that the analysis of ELISA-reactive and surface-reactive antibodies reveals different aspects of the antibody response. Indeed, although no surface cross-reactivity could be observed, there was clear evidence of cross-reactivity between the recombinant NTS-DBL1α_1_ domains in ELISA, with higher titres and values on the allelic forms than on the unrelated group C NTS-DBL1α. It appears that the group A/Cys2 DBL1α_1_ antigens studied here form a group of related serotypes, although more work is needed with additional domains from other subgroups to substantiate this conclusion. Accordingly, ELISA could potentially be used to define serogroups of related orthologs/alleles or paralogs. Similar ELISA cross-reactivity was reported with mouse or rabbit sera obtained by immunisation with recombinant domains from *var2csa* [34], [35], [38], [39] and from other rosette-forming parasites [41]. The strong inter-domain correlation and the similar age pattern of antibody acquisition observed here for the three group A NTS-DBL1α_1_ domains in the Senegalese community suggest that ELISA cross-reactivity occurs with human sera as well, although this was not formally tested by competition ELISA. Interestingly, limited intra-genome cross-reactivity of human antibodies with a panel of DBL1α and CIDR domains encoded by the 3D7 repertoire has been observed by competition ELISA but this was restricted to a few domains that shared more than 50% sequence identity [42]. This restricted inter-paralog cross-reactivity contrasts with the inter-allelic ELISA cross-reactivity observed here, and supports the interpretation that the three alleles studied form a serogroup of related serotypes. We suggest that ELISA detects more epitopes than surface immunofluorescence, agglutination or rosette disruption assays, perhaps by revealing conserved or shared epitopes of the protein that are not surface-accessible but become unveiled when coated on ELISA plates. In line with this, immunoblots of monovariant cultures showed variant specificity under non-reducing conditions but showed cross-reactivity under reducing conditions (data not shown). Antibodies reacting with linear conserved epitopes have been detected in sera from individuals living in areas where malaria is endemic [43], [44]. This probably accounts for the observation that ELISA reactivity with the recombinant domain correlated only moderately with iRBC surface reactivity. ELISA could thus be used to define serological subgroups of related orthologs/alleles or paralogs, but it does not predict surface reactivity and as such does not inform about potential selective forces driving surface polymorphism in the population. To study such selective forces - be they immunological or functional/adhesive - work with isolates from patients and with laboratory isolates should use monovariant, pure serotypes and adhesion types. Generating a panel of such variants will require a major community effort to derive the appropriate reagents.

The VarO, R29 and PF13 surface serotypes were quite frequent in the Senegalese community. They were acquired with different kinetics, the earliest response being apparently to VarO. Antibodies to R29 appear to be acquired later than antibodies to VarO or PF13. The low seroprevalence in young children is consistent with findings in Kenyan children [25]. Antibody acquisition in young children is consistent with a role for rosetting parasites in *falciparum* pathology, although children from Dielmo are usually protected against severe malaria from a very young age - earlier than the mean age for acquisition of surface-reacting antibodies, including antibodies to VarO. While a different study design is needed to analyse the potential contribution of the three variants to severe malaria, we can conclude that acquisition of specific antibodies coincides with mounting protecting response against malaria disease. Although, the precise mechanism of this immunity is still unclear, the present data are consistent with immunity against clinical malaria being brought about by the acquisition of a large repertoire of antibodies preventing expansion of a large number of antigenic variants.

We studied the domain-specific response using recombinant NTS-DBL1α_1_ domains that are correctly folded, as they met not only different biophysical criteria [25] but also induced surface reactive, rosette-disrupting antibodies and bound RBC [17], [40]. The antibody response acquired in endemic areas has been monitored using large panels of recombinant domains, although their native folding was usually not assessed and domain boundaries possibly not optimally designed. The definition of individual domains is not straightforward, as the original boundaries based on sequence alignment do not fully match with the crystal structures. In particular, our structural analysis of NTS-DBL1α_1_-varO.indicates that the NTS region is an intrinsic part of the domain. The recombinant DBL1α domains used by other groups to assess the acquired antibody response lacked the NTS moiety and in some instances did not include a critical last pair of cysteine residues [42], [45], [46], [47], [48]. It is therefore difficult to compare published seroprevalence for group A-derived domains with our findings. However, we note that the selevated seroprevalence against NTS-DBL1α_1_-R29 in Dielmo is in line with seroprevalence against DBL1α_1_-R29 in areas of high endemicity [49].

Of note, sequence identity of PF13 with VarO and R29 predicted the cytoadherence phenotype, as the three lines indeed formed dextran sulfate- and heparin-sensitive rosettes, suggesting that orthologs of NTS-DBL1α_1_-VarO in other parasite genomes are involved in rosetting as well. Our data also illustrate antigenic switching occurring in the IT4/R29 line, since after several months of culture including weekly enrichment for rosettes, surface reactivity to NTS-DBL1α_1_-R29 had dropped below 20%. This reflects switching expression from the dominant R29/*IT4var9* gene to another rosette-mediating paralog. Rosetting is encoded by a subset of *var* paralogs whose exact number is unknown. To circumvent the consequences of antigenic switching within the rosetting population in each of the three lines, we developed specific mAbs to pan the culture at regular intervals.

Surface seroreactivity to patient isolates has been extensively used to analyse the acquisition of responses [3], [16], [50], [51], [52], [53], [54], but these studies did not permit definition of specific serotype/serogroups as unpanned laboratory lines and patient isolates express multiple *var* genes [11], [55], [56], [57]. The same limitation applies to analysis of serotypes using lines selected for a specific cytoadherence because of the redundancy of cytoadherence specificities in the *var* repertoire [14], [58]. The only exception is *var2csa*, since it is single copy in most lines and the only gene associated with adhesion to CSA [59]; monovariant lines can be thus readily selected by panning on CSA. Apart from *var2csa*-expressing parasites, serology studies using monovariant lines are scarce. Analysis of seroreactivity of Kenyan sera to the monovariant A4u line panned using a specific mAb showed an age- and transmission-dependent acquisition of surface seroreactivity [47]. Interestingly, surface reactivity correlated poorly with ELISA reactivity to individual recombinant domains derived from the A4var gene expressed, consistent with our findings with the rosette-forming lines.

Both naturally acquired antibodies and mouse antibodies to the recombinant domains failed to show overlapping surface serotypes between the three related group A/Cys2 variants studied. This has major implications for vaccine development, as it indicates that specific strategies should be developed in order to target this particular subgroup considered serologically more conserved and cross-reactive than variants expressed in older children [42], [54] and thus of major interest for the development of a vaccine against severe *falciparum* malaria [13]. Such strategies could include the combining of multiple serotypes or engineering the immunogen to induce a broad reactivity. Both approaches face the challenge that we need a deeper understanding of the number of distinct genes implicated in rosetting and of their serogroup and serotype diversity. Mapping of the surface epitopes and the surface areas involved in adhesion will be facilitated by the recently determined crystal structure of the VarO adhesion domain; the available rosetting inhibitory anti-VarO mAbs should help this endeavour.

## Materials and Methods

### Production of NTS-DBL1α_1_ domains

Production of the VarO soluble recombinant domain has been described [25]. Recodoned R29 (Gencust, amino acids 1–481) and PF13 (GenScript; amino acids 1–486) domains had predicted N-glycosylation sites mutated to NxA to allow possible expression in eukaryotic systems and a C-terminal hexa-His tag. The coding sequence was cloned in pET21a (Novagen), and transformed into *E. coli* Rosetta-gami™ 2 (DE3) (Novagen). Protein expression was induced with 0.1 mM isopropyl-β-D-thio-galactopyranoside for 4 h at 30°C followed by 16 h at 20°C. Cells were resuspended in 20 mM Tris-HCl, 200 mM NaCl, pH 8 in presence of PMSF (Fluka) and Protease inhibitor cocktail complete EDTA-free (Roche) and lysed mechanically (EmulsiFlex-C5, Avestin). Soluble proteins were purified by metal affinity (TALON, Clontech) followed by size exclusion chromatography (Hiload 16/60 Superdex 200, GE Healthcare). The recodoned PFL1955w domain (without mutations or His tag) was cloned in pMAL-c2X (New England BioLabs) and expressed in *E. coli* SHuffle™ Express (New England BioLabs) as an MBP-fusion protein [25]. The protein was purified on an amylose column equilibrated with 20 mM Tris/HCl, pH 8, 200 mM NaCl, and released by passing the same buffer with 500 mM NaCl, 10 mM maltose. After size exclusion chromatography in the same Tris buffer, the fusion protein was cleaved with Factor Xa (Novagen) as described for the VarO domain. The domain was separated from MBP on a PD-10 column (GE Healthcare) in 20 mM Tris/HCl, pH 8, 50 mM NaCl, and a heparin column (HiTrap™ heparin HP, GE Healthcare) equilibrated with the same buffer. The domain was eluted with 20 mM Tris/HCl, pH 8, 300 mM NaCl.

### Production of mouse anti-NTS-DBL1α_1_ antibodies

Outbred mice were immunised as described [17]. Sera (N = 6 per group) were collected prior to the first injection (pre-immune sera) and 10 days after the third injection and stored at –20°C until use. R29-specific mouse mAbs were custom-produced (Genscript). PF13-specific mAbs were produced in-house as described [17]. MAbs were screened by ELISA on the immunising antigen and by iRBC surface reactivity. Animal studies complied with institutional guidelines of Institut Pasteur.

### Monovariant cultures

*P. falciparum* parasites were cultured in human O^+^ erythrocytes [17]. Late trophozoites/young schizont stages of IT4/R29 were weekly enriched for rosette-forming parasites by centrifugation on ice-cold Ficoll (density 1.077 mg.mL^−1^, Abcys). For 3D7 (obtained from MR4), weekly rosette enrichment for 2.5 months yielded a rosette-forming parasite culture. After rosette dissociation with 10 µg.mL^−1^ dextran sulfate (Sigma) incubated for 30 min at 37°C, mature iRBC were magnetically selected on a CS column (MACS, Miltenyi Biotec). For cell sorting, the iRBC pellet was resuspended in PBS, 2% foetal calf serum (FCS) and stained with anti-R29 or -PF13 sera (final dilution 1/200), and a goat anti-mouse IgG Alexa fluor 488 conjugate (dilution 1/1000). Surface-positive iRBCs were sorted by flow cytometry (MoFlo flow cytometer, Dako) and sub-cultured in O^+^ erythrocytes. Rosette formation was then selected by weekly enrichment and bi-monthly panning with specific mAbs as described [17]. Rosetting frequency and surface positivity in each monovariant culture was >90%.

### Human sera

Serum samples from 235 villagers (.5–85 years old) living in Dielmo (Senegal) were used. [17]. Non-immune sera from healthy adults living in France (Blood Bank, EFS, Rungis) were used as negative control.

### ELISA, surface immunofluorescence and rosette disruption assays

Experimental conditions for ELISA, surface immunofluorescence, flow cytometry and rosette-disruption assay using monovariant parasite cultures were as described [17].

### Mixed agglutination assays

After rosette disruption using dextran sulfate, iRBC were magnetically enriched. The iRBC membrane was labelled using PKH67 or PKH26 (Invitrogen) following the manufacturer's instructions, followed by three washes with PBS-2% FCS. Cells were resuspended in complete culture medium, 10 µg.mL^−1^ dextran sulfate. An equal volume of differentially stained iRBC was mixed and incubated 1 h at 37°C with human sera (final dilution 1/20) and Hoechst dye (dilution 1/1000). Immune agglutinates were visualised using fluorescent microscopy (Leica). An iRBC agglutinate was defined as a clump containing more than three iRBC. Each serum was tested in duplicate in two independent experiments.

### Depletion of human sera on recombinant NTS-DBL1α_1_ protein

10 µg of the VarO protein was absorbed on 10 µL of TALON resin (Clontech) for 1 h at 4°C. Unbound protein was removed by three washes with 20 mM Tris/HCl, pH 8, 200 mM NaCl. The resin was then incubated for 1 h at 4°C with human serum samples (4 µL of serum, 1/10 final dilution). The depleted sera were collected by centrifugation. Depletion was verified by ELISA.

### Mapping of surface-exposed polymorphic residues

The CONSURF web server [60] was used to assign a colour code according to the degree of conservation for each residue of the three variant sequences, which were aligned by ClustalW and corrected by hand, taking into account secondary structure elements of NTS-DBL1α_1_-varO. The result was displayed using PyMOL [61].

### Statistical analysis

ELISA OD and surface IFA values were log-transformed. The Student's t-test and Wilcoxon's nonparametric test were used to compare normal and non normal distributions of log-transformed data. Surface-reactive and domain-reactive antibodies were correlated using Spearman rank correlation test. Data were analyzed with STATA software (version 9.2, STATA corporation, College Station, TX, USA) and P<0.05 was considered to be statistically significant.

### Ethics statement

This study was carried out in strict accordance with the recommendations in the Guide for the Care and Use of Laboratory Animals of the Institut Pasteur (http://webcampus.pasteur.fr/jcms/c_98517/protocoles-usuels) and complied with the European Union guidelines for the handling of laboratory animals (http://ec.europa.eu/environment/chemicals/lab_animals/home_en.htm). The procedures were approved by the Institut Pasteur animal care and use committee. Animal care and handling was approved by the Ministère de l'Agriculture et de la Pêche (rapport 107503056792, clearance number 75–273, issued on 28th August 2007 to OMP) and the protocols and procedures used by the Direction départementale des services vétérinaires de Paris (Ref. RL- 07031395-30701147) isued on 29th August 2007 to OMP.

The human study was conducted in accordance with the Declaration of Helsinki and was approved by the Institutional Review Board of the Institut Pasteur of Paris (reference: RBm/2006.032 in September 2007) and by the National Council on Health Research of Senegal (reference n° 05 MSP/DS/CNRS in February 2008). Both review boards approved the verbal consent procedures. The project objectives were carefully explained to the assembled village population and the village head, the spokesperson for the women, for the younger generation, the religious authority of the village gave their informed consent. Individual verbal informed consent was then obtained, as most participants were illiterate, at each stage of the study. In formed consent was obtained individually from all participants (parents or ward of children) and was recorted in the individual villager's file. The possibility to withdraw from the project at any time was stated.

## Supporting Information

Figure S1 **PfEMP1 protein sequence analysis.** Pairwise amino acid sequence identity between domains of similar subtype from the Palo Alto/varO, IT4/R29, 3D7/PF13_0003 and 3D7/PFL1955w PfEMP1 proteins.
(PDF)

Figure S2 **SDS-Page analysis of the recombinant domains.** The recombinant NTS-DBL1α domains of VarO (lanes 1, 2), R29 (lanes 3, 4), PF13_0003 (lanes 5, 6) and PFL1955w (7,8) were migrated under reducing (even lanes) or non-reducing (odd lanes) conditions.
(PDF)

Figure S3 **Cross-reactivity between allelic domains measured by ELISA.** ELISA 50% and 95% titres of sera from mice immunised with individual recombinant NTS-DBL1α_1_ domains tested against the recombinant domains (top chart). Reduction factor of reactivity with the heterologous domains (bottom chart).
(PDF)

Figure S4 **Rosette-forming, surface-positive sorted iRBC used to derive IT4/R29 and 3D7/PF13_monovariant lines.** Microscopic visualisation (left) and immunostaining (right) of rosettes from the IT4/R29 (A) and 3D7/PF13_0003 (B) monovariant cultures obtained after cell sorting with mouse sera raised to the recombinant R29 and PF13_0003 domains respectively.
(PDF)
