# Supplementary material for: Allelic Diversity of the Plasmodium falciparum Erythrocyte Membrane Protein 1 Entails Variant-Specific Red Cell Surface Epitopes
Source: PLoS One. 2011 Jan 27;6(1):e16544. doi: 10.1371/journal.pone.0016544 (PMC3029348; doi:10.1371/journal.pone.0016544)
Supplement: Figure S1 — PfEMP1 protein sequence analysis. Pairwise amino acid sequence identity between domains of similar subtype from the Palo Alto/varO, IT4/R29, 3D7/PF13_0003 and 3D7/PFL1955w PfEMP1 proteins. (PDF) [file pone.0016544.s001.pdf]

Query domain	NTS-DBL1α varO	NTS-DBL1α R29	NTS-DBL1α PF13_0003
**NTS-DBL1α1**			
NTS-DBL1α R29	60.5		
NTS-DBL1α PF13_0003	52.7	48.4	
NTS-DBL1α PFL1955w	29.2	32.9	32.9

**CIDRγ**	CIDRγ varO	CIDR1γ R29
CIDRγ varO		
CIDRγ R29	26.9	
CIDRγ PF13_003	24.0	28.3

**DBLβ**	DBL2β varO	DBL4β varO	DBL5β varO	DBL2β PF13_0003
DBL2β varO				
DBL4β varO	22.4			
DBL5β varO	17.1	17.4		
DBL2β PF13_0003	38.3	22.6	15.3	
DBL5β PF13_0003	38.1	20.3	17.4	38.3

**DBLγ**	DBL3γ varO	DBL3γ R29
DBL3γ varO		
DBL3γ R29	25.1	
DBL3γ PF13_003	28.9	26.4

**DBLδ**	DBL4δ R29
DBL4δ R29	
DBL4δ PF13_0003	27.2

**CIDRβ**	CIDRβ R29
CIDRβ R29	
CIDRβ PF13_0003	25.6
