# Supplementary figures and images for: Allelic Diversity of the Plasmodium falciparum Erythrocyte Membrane Protein 1 Entails Variant-Specific Red Cell Surface Epitopes
Source: PLoS One. 2011 Jan 27;6(1):e16544. doi: 10.1371/journal.pone.0016544 (PMC3029348; doi:10.1371/journal.pone.0016544)

**1****2****3****4****5****6****7****8****250****150****100****75****50****37****25****20**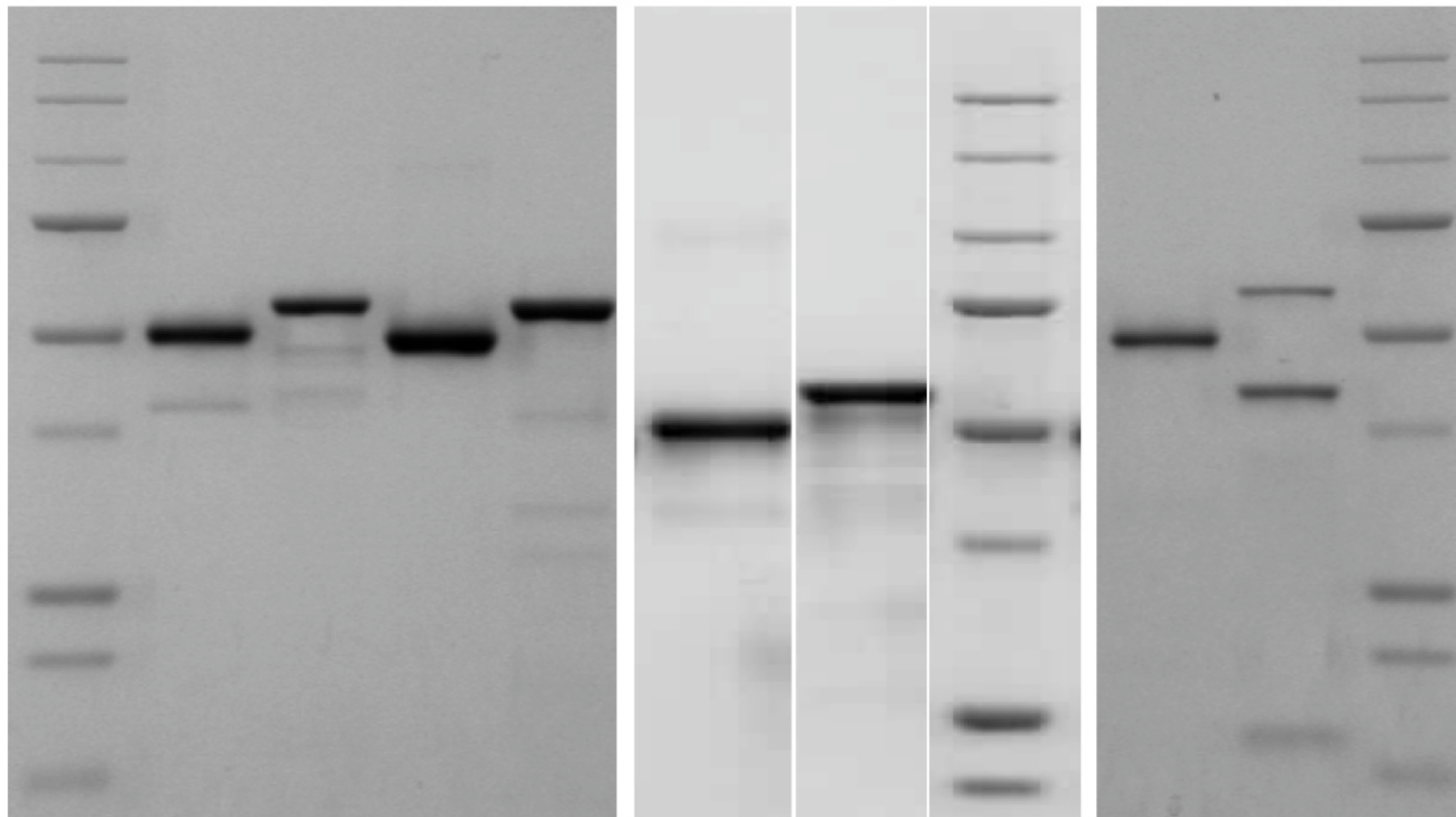

Supplement: Figure S2 — SDS-Page analysis of the recombinant domains. The recombinant NTS-DBL1α domains of VarO (lanes 1, 2), R29 (lanes 3, 4), PF13_0003 (lanes 5, 6) and PFL1955w (7,8) were migrated under reducing (even lanes) or non-reducing (odd lanes) conditions. (PDF) [file pone.0016544.s002.pdf]

**A**

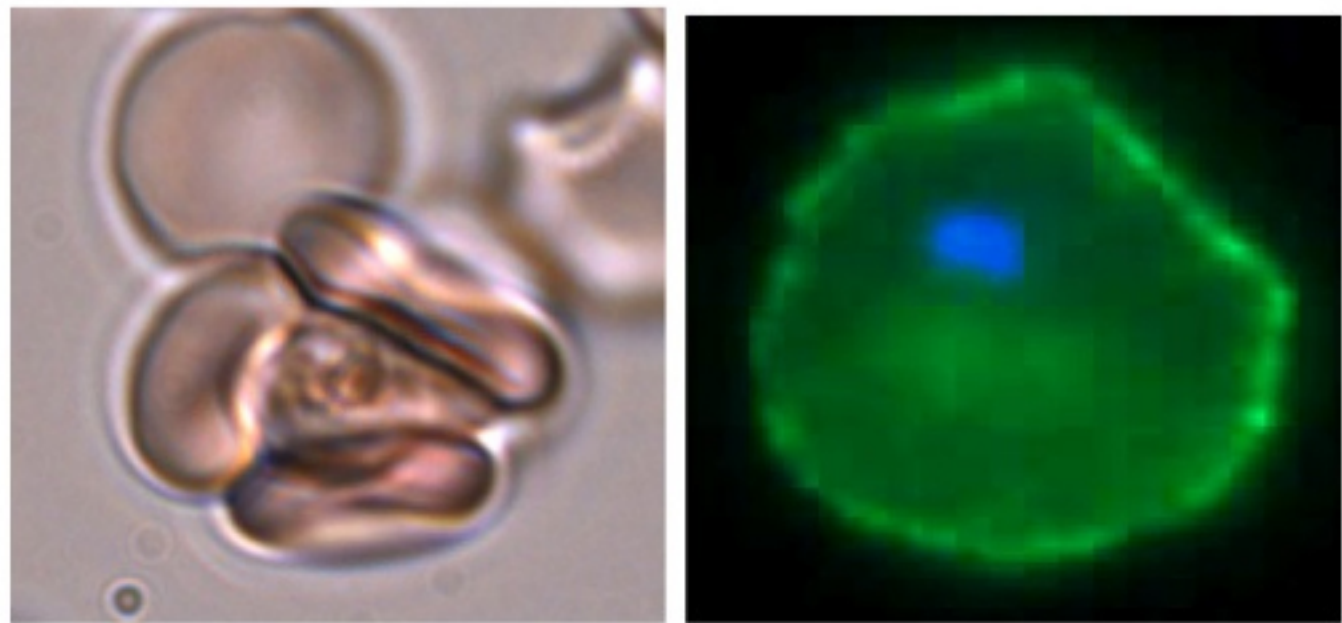

**R29 iRBC**

**B**

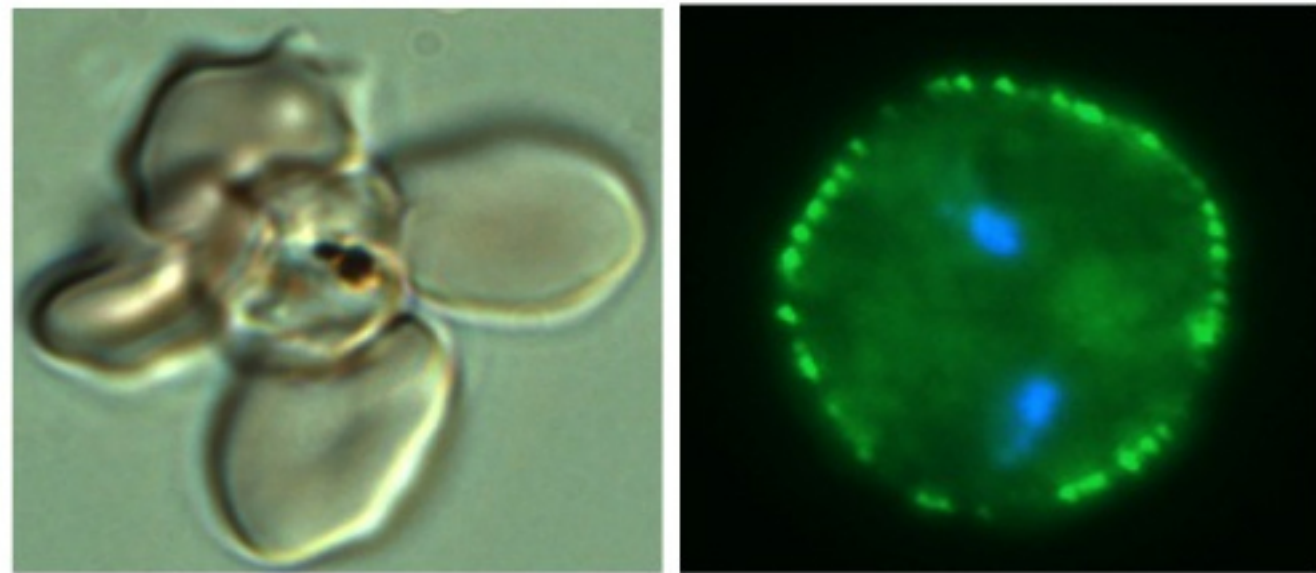

**PF13 iRBC**

Supplement: Figure S4 — Rosette-forming, surface-positive sorted iRBC used to derive IT4/R29 and 3D7/PF13_monovariant lines. Microscopic visualisation (left) and immunostaining (right) of rosettes from the IT4/R29 (A) and 3D7/PF13_0003 (B) monovariant cultures obtained after cell sorting with mouse sera raised to the recombinant R29 and PF13_0003 domains respectively. (PDF) [file pone.0016544.s004.pdf]
