# Supplementary material for: Allelic Diversity of the Plasmodium falciparum Erythrocyte Membrane Protein 1 Entails Variant-Specific Red Cell Surface Epitopes
Source: PLoS One. 2011 Jan 27;6(1):e16544. doi: 10.1371/journal.pone.0016544 (PMC3029348; doi:10.1371/journal.pone.0016544)
Supplement: Figure S3 — Cross-reactivity between allelic domains measured by ELISA. ELISA 50% and 95% titres of sera from mice immunised with individual recombinant NTS-DBL1α1 domains tested against the recombinant domains (top chart). Reduction factor of reactivity with the heterologous domains (bottom chart). (PDF) [file pone.0016544.s003.pdf]

	50% titer				endpoint titer (95%)			
	**VarO**	**R29**	**PF13**	**PFL**	**VarO**	**R29**	**PF13**	**PFL**
**Anti-VarO**	**409 600**	4 000	10 00	1 000	**6 553 600**	204 800	409 600	6 400
**Anti-R29**	4 000	**204 800**	5 000	2 000	102 400	**3 276 800**	40 000	12 800
**Anti-PF13**	1 000	2 000	**204 800**	2 500	204 000	75 000	**3 276 800**	25 600

	50% titer				endpoint titer (95%)			
	**VarO**	**R29**	**PF13**	**PFL**	**VarO**	**R29**	**PF13**	**PFL**
**Anti-VarO**	**409 600**	100-fold	40-fold	400-fold	**6 553 600**	30-fold	16-fold	1000-fold
**Anti-R29**	50-fold	**204 800**	40-fold	100-fold	30-fold	**3 276 800**	80-fold	250-fold
**Anti-PF13**	20-fold	100-fold	**204 800**	80-fold	16-fold	45-fold	**3 276 800**	128-fold
